# Beyond biochemistry: multiparametric ultrasound parameters and their molecular correlates in cardio-renal-metabolic syndrome

**DOI:** 10.3389/abp.2026.16873

**Published:** 2026-07-14

**Authors:** Marcin L. Kutek, Jacek M. Witkowski

**Affiliations:** 1 Department of Embryology, Medical University of Gdańsk, Gdańsk, Poland; 2 Department of Physiopathology, Medical University of Gdańsk, Gdańsk, Poland; 3 Department of Hypertension and Diabetology, Medical University of Gdańsk, Gdańsk, Poland

**Keywords:** cardio-renal-metabolic syndrome, carotid intima-media thickness, elastography, epicardial adipose tissue, multiparametric ultrasound

## Abstract

Cardio-renal-metabolic syndrome (CRMS)—characterized by the pathological interplay of visceral adiposity, insulin resistance, chronic kidney disease, and cardiovascular disease—affects over 90% of US adults across its staging spectrum, yet its multi-organ burden remains difficult to assess non-invasively at the point of care. This narrative mini review examines whether multiparametric ultrasound within a single examination can serve as an integrated imaging biomarker set complementary to established molecular markers of CRMS. A narrative search of PubMed (2015–2026) was conducted using PICO-structured queries. The hepatic controlled attenuation parameter and liver stiffness measurement correlate directly with Homeostatic Model Assessment of Insulin Resistance (HOMA-IR), CRP, and adipokine dysregulation, with pooled CAP cutoffs of 268–313 dB/m and elastography thresholds of 8.2–13.6 kPa for fibrosis staging in MASLD. Epicardial adipose tissue thickness correlates with circulating IL-17A (r = 0.308), hs-CRP (r = 0.666), and HOMA-IR (r = 0.567–0.580), independently predicting left ventricular diastolic dysfunction beyond BMI. Carotid intima-media thickness tracks eGFR decline longitudinally and predicts cardiovascular mortality in CKD populations. Renal resistive index, with a validated threshold of ≥0.70, independently predicts GFR decline and cardiovascular mortality across diabetic and hypertensive populations. Renal shear wave elastography distinguishes fibrotic from non-fibrotic parenchyma, with 93.1% sensitivity against biopsy. Cross-compartmental correlations and composite imaging-biochemistry models consistently outperform single-parameter approaches. Two critical gaps remain: specific ultrasound values have not been associated with AHA/ACC CKM stages, and no outcome study has validated a multiorgan protocol. Multiparametric ultrasound provides a clinically feasible, evidence-based CRMS assessment that remains to be validated in prospective trials.

## Introduction

Cardio-renal-metabolic syndrome (CRMS) is defined by the pathophysiological interplay of metabolic risk factors, excess adiposity, and cardiovascular and chronic kidney disease, recognized in the 2023 American Heart Association presidential advisory as a four-stage continuum ranging from metabolic risk factors (stage 1) to established cardiovascular disease with concurrent kidney or metabolic disorder (stage 4) (Ndumele et al., 2023a; Ndumele et al., 2023b). Approximately 90% of adults fall within CRMS stages 1–4, underscoring the need for accessible tools to identify the multi-organ disease process (Gunnarsson et al., 2025; Lei et al., 2025). Current assessment relies on biochemical markers that capture the systemic molecular milieu but do not directly evaluate organ-level structural consequences. Ultrasound is uniquely positioned to bridge this gap; it is non-ionizing, available at the point-of-care, and capable of assessing hepatic, cardiac, vascular, and renal compartments within a single examination.

This review addresses the following question: In adult patients with CRMS (P), can multiparametric ultrasound assessment of hepatic, visceral, cardiac, vascular, and renal parameters (I) serve as an integrated imaging biomarker complementary to established molecular and biochemical markers (C), supporting its use as a first-line screening tool for CRMS staging and risk stratification (O)?

A narrative search of PubMed and the Consensus AI-assisted academic search engine (consensus.app) (January 2015 – April 2026) was conducted using PICO-structured queries targeting each organ compartment and molecular aspect reviewed. Peer-reviewed publications in English reporting original data, systematic reviews, or meta-analyses in human populations were prioritized. To mitigate potential AI search bias, complementary technique-oriented and phenotype-oriented PICO queries were formulated for each topic, and Consensus results were verified against PubMed records; full-text screening was performed by the primary author for all cited studies. Data extraction was performed by the first author from full-text articles using NotebookLM (notebooklm.google.com). The complete search parameters are provided in Supplementary Material 1. All content was critically reviewed and approved by both authors prior to submission.

## Molecular landscape of CRMS

CRMS arises from dysfunctional visceral adipose tissue, whose expansion drives chronic low-grade inflammation, insulin resistance, and inappropriate endocrine function—pathophysiological processes that each ultrasound parameter in this review reflects at the organ-structural level (Neeland et al., 2019; Ndumele et al., 2023b).

### Adipokine dysregulation

Serum concentrations of leptin, resistin, and visfatin, the pro-inflammatory adipokines, are elevated, which promotes the development of insulin resistance, atherogenesis, and sympathetic activation. Concurrently, the concentration of protective adipokines, namely adiponectin and omentin, is decreased (Recinella et al., 2020; Aljafary and Al-Suhaimi, 2022; Gupta et al., 2023). The adiponectin/leptin ratio correlates with the development of insulin resistance and cardiovascular comorbidities (Frühbeck et al., 2018; Fajkić et al., 2024). A ratio below 0.5 indicates severe cardiometabolic risk, while above 1.0 is considered normal (Frühbeck et al., 2018). Paradoxically, both adiponectin and leptin rise in advanced CKD due to reduced renal clearance; elevated adiponectin predicts protein-energy wasting and cardiovascular mortality (Coimbra et al., 2022).

### Chronic low-grade inflammation

Altered adipose tissue sustains chronic macrophage-driven inflammation (Mouton et al., 2020; Nzobokela et al., 2025). Across the CRMS spectrum, IL-6, TNF-α, and CRP are elevated, and their serum levels serve as predictors of myocardial fibrosis, CKD progression, and major cardiovascular events (Xu et al., 2025). Moreover, co-occurrent CRP and IL-6 elevation confers a 5.1-fold increased risk of developing type 2 diabetes, underscoring the bidirectional relationship between inflammation and metabolic deterioration (Lainampetch et al., 2019). NLRP3 inflammasome activation by metabolic danger signals drives IL-1β maturation, exacerbating atherosclerosis and renal fibrosis (Nzobokela et al., 2025; Xu et al., 2025). Cardiometabolic disorders associated with the monocyte subset shift toward pro-inflammatory phenotypes and CD8^+^ T cell activation correlating with fasting glucose, sustaining the vascular and renal inflammatory cascade (Marques et al., 2019).

### Insulin resistance indices

HOMA-IR remains the most widely used clinical surrogate for insulin resistance, with proposed sex-specific cutoffs of 2.00 for male and 2.50 for female patients with metabolic syndrome (Netala et al., 2025). The triglyceride-glucose (TyG) index and TyG-BMI are validated alternatives; a ten-unit increase in TyG-BMI associates with a 6.5% higher risk of incident cardiovascular disease in CKM populations (Xu et al., 2025).

### Kidney injury and function markers

Tubular injury biomarkers—cystatin C, NGAL, and KIM-1—detect structural damage earlier than eGFR, while albuminuria assessed by the urine albumin-to-creatinine ratio serves as a marker of renal microvascular damage and systemic vascular stress across all CRMS stages (Nzobokela et al., 2025).

## Multiparametric ultrasound in CRMS assessment

Multiparametric ultrasound is defined as advanced imaging that combines various ultrasonographic parameters into a single examination.

### Visceral fat and hepatic and pancreatic steatosis

Once subcutaneous adipose tissue reaches its storage capacity, the lipids overflow into ectopic storage depots, where they can then acquire pro-inflammatory secretory profiles (Neeland et al., 2019). This pathophysiologically interconnected process results in an anatomically heterogeneous fat distribution across visceral, hepatic, epicardial, and pancreatic compartments. Structured ultrasound assessment of omental fat at the L4 level demonstrates strong correlations with HOMA-IR (r = 0.279), fasting glycaemia (r = 0.380), and HbA1c (r = 0.232), while the peri-renal fat compartment correlates inversely with HDL cholesterol (Di Gregorio et al., 2026). Across advancing CKM syndrome stages, epicardial and peri-renal fat depots increase progressively in parallel with insulin resistance, whereas subcutaneous fat does not, demonstrating that VAT-targeted sonographic assessment carries metabolic information that waist circumference and BMI alone cannot provide (Neeland et al., 2019; Cho et al., 2025).

The liver provides the most extensively studied and validated site for ectopic fat assessment. Using a transient elastography probe (FibroScan), the controlled attenuation parameter (CAP) quantifies hepatic fat content with pooled cutoffs of approximately 268 dB/m for S ≥ 1, 288 dB/m for S ≥ 2, and 313 dB/m for S ≥ 3 steatosis across large individual patient data meta-analyses (Petroff et al., 2021; Cao et al., 2022). CAP values differ by 15–24 dB/m between M and XL probes, with a discrepancy sufficient to cause misclassification at the individual level (Petroff et al., 2021). CAP correlates directly with insulin resistance: patients with HOMA-IR above 2.5 carry a 3.21-fold risk of elevated CAP (Petralli et al., 2023), while HOMA-IR independently correlates with both CAP (r = 0.36) and B-mode steatosis grade (r = 0.44) (Stepanov et al., 2022). Liver steatosis additionally correlates with CRP concentrations (r = 0.233) and FGF-21 (r = 0.313), linking ultrasound-detectable fat accumulation to the systemic inflammatory and endocrine milieu (Cantero et al., 2019). Liver stiffness correlates independently with HOMA-IR (r = 0.275) and fasting insulin (r = 0.270) regardless of BMI and transaminases, confirming that hepatic stiffening reflects insulin-driven portal inflammation rather than merely the mechanical consequence of fat deposition (Petralli et al., 2023).

TE measures visco-elasticity and may overestimate stiffness in active hepatic inflammation, whereas 2D-SWE isolates the elastic component and is less confounded by necro-inflammatory activity (Mendoza et al., 2021); two-dimensional attenuation imaging (2D-ATI) is emerging as a more reproducible successor to CAP for hepatic fat quantification (Hobeika et al., 2024; Nishimura et al., 2025).

Pancreatic steatosis, conceptualized as metabolic dysfunction-associated steatotic pancreas disease (MASPD), is assessed using ultrasound by comparing pancreatic echogenicity to that of the renal cortex and retroperitoneal fat (Ulaşoğlu et al., 2021). It has been found that severe MASPD correlates with insulin resistance markers, with reported odds ratios of 6.20 for HOMA-IR and 5.72 for the TyG index (De Oliveira Andrade et al., 2024). The principal limitation of ultrasound in this context is its inability to differentiate true intralobular parenchymal fat from peripancreatic visceral fat (Begovatz et al., 2015).

### Vascular parameters: carotid intima-media thickness and epicardial adipose tissue

Carotid IMT, measured according to Mannheim Consensus Guidelines at the far wall of the common carotid artery, serves as an established marker; values exceeding 0.9 mm indicate asymptomatic organ damage (Deepa et al., 2025). In patients with type 2 diabetes and CKD, a cIMT above 0.86 mm independently predicts all-cause mortality (HR 2.9) and cardiovascular events (HR 2.04) after adjustment for eGFR and albuminuria (Roumeliotis et al., 2019). Also, among hemodialysis patients, a per-unit increase carries a relative risk of 1.08 for all-cause and 1.29 for cardiovascular mortality (Janda et al., 2015; Kouis et al., 2019).

cIMT correlates strongly with the concentration of osteopontin, an inhibitor of vascular calcification (Chaitanya et al., 2018). A four-year longitudinal study demonstrated that progressive eGFR decline in CKD stage 4 directly parallels progressive cIMT expansion from 1.13 to 1.25 mm, establishing cIMT as a dynamic imaging marker of the renal-vascular axis crucial to CRMS pathophysiology (Rizikalo et al., 2021). The presence of a carotid plaque, defined as focal intimal protrusion exceeding 0.5 mm or a thickness above 1.5 mm, is more predictive of CKD progression and cardiovascular events than common carotid artery IMT alone (Ohtake and Kobayashi, 2020).

Epicardial adipose tissue differs from other fat deposits by having a direct anatomical continuity with the myocardium and coronary vessels, exerting vasocrine and paracrine effects via shared microcirculation; it is measured as the echo-free space on the right ventricular free wall at end-systole by transthoracic echocardiography (Iacobellis, 2015; Ansaldo et al., 2019). Proposed risk thresholds range from 4.7 mm for metabolic syndrome (sensitivity 77.5% and specificity 87.5%) to 7.5–9.5 mm for high cardiometabolic risk stratified by sex (Patel et al., 2017; Demir et al., 2019). EAT secretes IL-6, TNF-α, and IL-17A into the coronary vasa vasorum, with thickness correlating with circulating IL-17A (r = 0.308), hs-CRP (r = 0.666), and HOMA-IR (r = 0.567–0.580) (Singh et al., 2018; Demir et al., 2019). A systematic review and meta-analysis by Zhong-Yan confirmed that EAT volume and thickness are significantly greater in patients with metabolic syndrome independently of conventional risk markers (Zhong-Yan et al., 2023), and EAT thickness constitutes an independent predictor of left ventricular diastolic dysfunction after adjusting for BMI and waist circumference (Cho et al., 2025). Interestingly, GLP-1 receptor agonists reduce EAT thickness by approximately 7%–8% independently of weight loss, and SGLT-2 inhibitors reduce EAT to an even greater extent than either GLP-1 agonists or exercise interventions, which makes EAT an attractive target for both diagnosis and therapeutic monitoring parameter in CRMS (Myasoedova et al., 2023; Bao et al., 2024). Elevated EAT and its paracrine dysfunction correlate with subclinical atherosclerosis, including increased cIMT, positioning these two parameters as complementary rather than redundant within a vascular ultrasound protocol (Iacobellis, 2022; Krishnan et al., 2022).

### Renal parameters: resistive index, cortical echogenicity, and elastography

RRI, obtained by pulsed-wave Doppler at interlobar arteries, with a threshold of ≥0.70 predicts abnormal renal vascular resistance and has been found to predict both cardiovascular mortality and CKD progression among diabetic, heart failure, and hypertensive populations (Boddi, 2016; Provenzano et al., 2020; Romano et al., 2022; Kuttancheri et al., 2023). Provenzano et al. have also proposed a model in which an RRI of 0.65 detects early tubular and glomerular injury, while ≥0.70 correlates with future eGFR decline (Provenzano et al., 2020). Interestingly, a per 0.1-unit increment predicts five-year renal disease progression and long-term mortality in non-proteinuric CKD patients (Romano et al., 2022; 2025). In populations at risk of CRMS, those suffering from diabetes present higher mean RRI (0.72) than non-diabetic controls (0.65), and the development of microalbuminuria is preceded by an elevated RRI above 0.70 (Boddi, 2016; Barone et al., 2024).

Renal cortical echogenicity may reflect tubulointerstitial fibrosis, glomerular sclerosis, and tubular atrophy. When assessed by the Brenbridge echogenicity grading system, it demonstrates higher specificity (96%) and positive predictive value (75%) for CKD staging than RRI, though limited by poor inter-observer reproducibility (Sutikno and Baskoro, 2020). The 2024 KDIGO Guideline reinforces imaging integration with structural and functional biomarkers for CKD risk stratification (Stevens et al., 2024).

Perirenal adipose tissue, measured by B-mode ultrasound, exerts deleterious paracrine effects through TNF-α, IL-6, leptin, and renin-angiotensin-aldosterone system activation (Qiu et al., 2024). It was shown to predict CKD in T2DM patients (HR 1.67 per SD increment) and renal SWE stiffness at CKD stage 3 (Chen et al., 2021; Xu et al., 2023; Li et al., 2024) as well as negatively correlate with eGFR among all adiposity indices (r = −0.29 to −0.43, AUC 0.686), thus mechanistically linking ectopic lipotoxicity with parenchymal fibrosis.

Renal SWE distinguishes fibrotic from non-fibrotic parenchyma with a cortical stiffness cutoff of 4.05 kPa when compared with kidney biopsy (Zhang et al., 2024), whereas across mild, moderate, and severe fibrosis grades, pooled AUROC values of 0.87, 0.78, and 0.86, respectively, have been reported (Cao et al., 2023). The renal stiffness was also found to inversely correlate with eGFR (r = −0.329 to −0.65) across diabetic nephropathy, nephrosclerosis, and glomerulonephritis etiologies (Kuttancheri et al., 2023; Filipov et al., 2024). When applied in patients with metabolic syndrome, the multiparametric ultrasound phenotyping yields five nephropathy patterns relevant to CRMS, namely diabetic (thinned parenchyma, RI > 0.7), ischemic-atherosclerotic (reduced kidney size, RI > 0.8, flow velocities <25 cm/s), hypertensive (elevated RI and preserved morphology), gout-associated (hyperechoic inclusions, hilly cortical margins, SWE 8.7–10.0 kPa), and the circular pyramids pattern (hypoechoic perimedullary rings), which are each linked to a distinct molecular axis (Bubnov, 2023). In biopsy-validated phenotyping in diabetic CKD, RI was shown to discriminate pure metabolic from vascular nephropathy (p = 0.02) (Claudel et al., 2025). The sonographic gout nephropathy pattern is further supported by a cross-sectional study demonstrating that hyperechoic kidney medullary deposits in gouty subjects were associated with eGFR decline and tubulointerstitial nephritis on histology (Bardin et al., 2021). While the evidence base for the five-pattern CRMS nephropathy classification remains heterogenous in study design and scale, the convergent findings across independent methodologies support the rationale for prospective multicenter validation studies.

Another important phenotype, post-COVID-19 nephropathy–driven by endothelial injury and microvascular thrombosis–was shown to be detectable by ultrasound: SWE stiffness reaching 10 ± 1.7 kPa in severe and 7.2 ± 1.5 kPa in moderate post-COVID renal injury versus 4.2 ± 1.2 kPa in controls, with concomitant RRI >0.75, cortical thinning, and hyperechoic inclusions (Bubnov, 2022). Nonetheless, the SWE interpretation needs to account for confounding factors specific to the CRMS population: kidney depth exceeding 5 cm, a hemodynamic state, tissue anisotropy, and metabolic inflammation (Maralescu et al., 2022; Qiang et al., 2024; Albrizio, 2025). Moreover, renal SWE cutoffs are not yet standardized across ultrasound vendors nor have they been validated in large CRMS-specific cohorts, limiting their direct translation into the clinical settings and precluding definitive diagnostic thresholds at this stage (Cao et al., 2023; Kuttancheri et al., 2023).

Taken together, these renal parameters allow for the assessment of renal phenotyping with multiple parameters, such as morphological, hemodynamic, ectopic metabolic, and rheological, with capillary-level microvascular assessment discussed in Section *Emerging techniques: contrast-enhanced ultrasound and microvascular imaging in CRMS nephropathy phenotypes*. The key ultrasound parameters discussed in Sections *Visceral fat and hepatic and pancreatic steatosis, Vascular parameters: carotid intima-media thickness and epicardial adipose tissue, Renal parameters: resistive index, cortical echogenicity, and elastography*, together with their molecular correlates, proposed cutoffs, and clinical significance in CKM syndrome, are summarized in Table 1.

**TABLE 1:** Multiparametric ultrasound parameters in cardio-renal-metabolic syndrome: molecular correlates, diagnostic cutoffs, clinical significance, and translational readiness.

Ultrasound parameter	Target organ	Key molecular correlates	Proposed cutoff(s)	Clinical significance in CKM syndrome	Key references	Clinical applicability
Omental/visceral fat thickness (B-mode)	Visceral adipose tissue	HOMA-IR (r = 0.28), HbA1c (r = 0.23), fasting glucose (r = 0.38), HDL↓	No validated US cutoff; correlational data only	Tracks insulin resistance across CKM stages; VAT-specific metabolic signal not captured by BMI or waist circumference	Neeland et al. (2019), Di Gregorio et al. (2026), Cho et al. (2025)	B Specialised centres
Controlled Attenuation Parameter (CAP)	Liver	HOMA-IR (3.21× risk >2.5), CRP (r = 0.23), FGF-21 (r = 0.31)	S ≥ 1: 268 dB/m; S ≥ 2: 288 dB/m; S ≥ 3: 313 dB/m (M probe); M vs. XL probe discrepancy: 15–24 dB/m	Non-invasive hepatic steatosis grading reflecting insulin resistance burden; probe-dependent — M vs. XL probe causes clinically relevant misclassification	Petroff et al. (2021), Cao et al. (2022), Petralli et al. (2023), Stepanov et al. (2022)	A Routine practice
Liver Stiffness Measurement (LSM; TE/2D-SWE)	Liver	HOMA-IR (r = 0.28), fasting insulin (r = 0.27); independent of BMI and transaminases	F ≥ 2: ∼7.9 kPa; F ≥ 3: ∼9.6 kPa; cirrhosis: ≥13.6 kPa (MASLD, TE)	Reflects insulin-driven portal inflammation; TE overestimates stiffness in active inflammation — 2D-SWE preferred in CRMS; Agile score predicts incident CKD	Petralli et al. (2023), Mendoza et al. (2021), Eddowes et al. (2019), Jung et al. (2024)	A Routine practice
Pancreatic echogenicity (B-mode; MASPD)	Pancreas	HOMA-IR (OR 6.20), TyG index (OR 5.72)	Qualitative: hyperechoic parenchyma vs. renal cortex and retroperitoneal fat	Metabolic dysfunction-associated steatotic pancreas disease; cannot distinguish intralobular from peripancreatic fat; correlates with beta-cell dysfunction	Ulaşoğlu et al. (2021), De Oliveira Andrade et al. (2024), Begovatz et al. (2015)	B Specialised centres
Epicardial Adipose Tissue thickness (EAT; echocardiography)	Pericardium/Coronary vasculature	IL-17A (r = 0.31), hs-CRP (r = 0.67), HOMA-IR (r = 0.57–0.58); secretes IL-6, TNF-α	MetS risk: ≥4.7 mm (Sn 77.5%, Sp 87.5%); high cardiometabolic risk: 7.5–9.5 mm (sex-stratified)	Independent predictor of LV diastolic dysfunction; reduced by SGLT-2i > GLP-1 RA; complementary to cIMT for subclinical atherosclerosis	Demir et al. (2019), Singh et al. (2018), Zhong-Yan et al. (2023), Myasoedova et al. (2023), Bao et al. (2024), Cho et al. (2025)	B Specialised centres
Carotid Intima-Media Thickness (cIMT)	Carotid artery	Osteopontin (inhibitor of vascular calcification); tracks eGFR decline longitudinally	Organ damage: >0.9 mm; mortality in T2DM + CKD: >0.86 mm; plaque: protrusion >0.5 mm or thickness >1.5 mm	Predicts all-cause mortality (HR 2.9) and CV events (HR 2.04) in T2DM + CKD; RR 1.08–1.29 per-unit in HD patients; dynamic marker of renal-vascular axis	Roumeliotis et al. (2019), Janda et al. (2015), Kouis et al. (2019), Rizikalo et al. (2021), Ohtake and Kobayashi (2020)	B Specialised centres
Carotid Plaque Grey-Scale Median (GSM)	Carotid artery	MMP-9, MMP-3, TIMP-1 (extracellular matrix remodelling)	Low GSM + elevated MMP-9 predicts MACE; no single numerical cutoff validated	Plaque vulnerability assessment; combined with NT-proBNP, hs-cTNT, hs-CRP yields 8-fold cardiovascular risk gradient over 3 years	Jiao et al. (2020), Kadoglou et al. (2023), Gore et al. (2020)	C Research stage
Perirenal Fat Thickness (PRFT; B-mode)	Perirenal adipose tissue	TNF-α, IL-6, leptin, RAAS hyperactivation; eGFR (r = −0.29 to −0.43)	No validated US cutoff; HR 1.67/SD increment for incident CKD in T2DM (CT-based data)	Strongest adiposity predictor of eGFR decline (AUC 0.69); increases with CKM stages parallel to EAT; independent predictor of renal SWE stiffness at CKD stage 3	Qiu et al. (2024), Chen et al. (2021), Xu et al. (2023), Li et al. (2024), Cho et al. (2025)	C Research stage
Renal Resistive Index (RRI; pulsed-wave Doppler)	Kidney (interlobar arteries)	eGFR (inverse), serum phosphate, diabetes status, tubular injury markers	≥0.70: abnormal vascular resistance, predicts CKD progression and CV mortality; ≥0.65: early tubular/glomerular injury	Mean RRI 0.72 in CRMS-diabetes vs. 0.65 in controls; each 0.1-unit increment predicts 5-year renal disease progression and long-term mortality	Boddi (2016), Provenzano et al. (2020), Romano et al. (2022), Romano et al. (2025), Kuttancheri et al. (2023), Barone et al., 2024	A Routine practice
Renal Cortical Echogenicity (B-mode; Brenbridge scale)	Kidney (cortex)	Serum creatinine/eGFR (r = 0.84–0.91); calcium-phosphate metabolism	Qualitative grading (0–3); grade ≥2 corresponds to CKD; specificity 96%, PPV 75% vs. RRI	Reflects tubulointerstitial fibrosis, glomerular sclerosis, tubular atrophy; high specificity but poor inter-observer reproducibility	Ahmed et al. (2019), Sutikno and Baskoro (2020), Stevens et al. (2024)	B Specialised centres
Renal Shear Wave Elastography (SWE)	Kidney (cortex)	HbA1c (r = 0.53), uric acid (r = 0.48), eGFR (r = −0.33 to −0.65); interstitial fibrosis score (r = 0.76)	Fibrosis cutoff: 4.05 kPa; CKD stage 1→5: ∼4.8→10.8 kPa; metabolic phenotype: 7.2 ± 1.1 kPa; gout nephropathy: 8.7–10.0 kPa	Phenotypes 6 CRMS nephropathy patterns including post-COVID (SWE 10 ± 1.7 kPa); cutoffs not standardised across platforms; confounded by kidney depth >5 cm, hemodynamics, anisotropy, metabolic inflammation	Cao et al. (2023), Zhang et al. (2024), Kuttancheri et al. (2023), Filipov et al. (2024), Bubnov (2022), Bubnov (2023); Li et al. (2024)	C Research stage
Superb Microvascular Imaging (SMI)/CEUS Vascular Index	Kidney (cortical microvasculature)	eGFR (r = 0.56, SMI), biopsy-confirmed fibrosis; CEUS PI lower in vascular vs. diabetic nephropathy; BNP at discharge (r = 0.57, IRPI)	SMI index: no validated cutoff; lower in CKD vs. controls (49.9% vs. 72.2%); CEUS PI: 1618 vs. 3176 au (CKD vs. controls); IRPI: AUC 0.73 for CV endpoints	Capillary-level assessment without nephrotoxicity; IRPI predicts CV endpoints in cardiorenal CRMS; phenotype-specific CEUS PI differentiates vascular from diabetic nephropathy; emerging role in post-COVID renal injury (see Section *Emerging techniques: contrast-enhanced ultrasound and microvascular imaging in CRMS nephropathy phenotypes*)	Armaly et al. (2022), Kayama (2024), Kayama (2025); Querfeld et al. (2020)	C Research stage

Clinical Applicability tiers: A (green) = Available in routine practice (guideline-endorsed, validated cutoffs, widely accessible); B (yellow) = Feasible in specialised centres (requires dedicated expertise or equipment, not universally guideline-endorsed); C (orange) = Currently research stage (no standardised cutoffs, requires prospective multicentre validation before clinical adoption).

Abbreviations: AUC, area under the curve; au, arbitrary units; CAP, controlled attenuation parameter; CEUS, contrast-enhanced ultrasound; cIMT, carotid intima-media thickness; CKD, chronic kidney disease; CKM, cardiovascular-kidney-metabolic; CV, cardiovascular; EAT, epicardial adipose tissue; eGFR, estimated glomerular filtration rate; GSM, grey-scale median; HbA1c, glycated haemoglobin; HD, haemodialysis; HOMA-IR, Homeostatic Model Assessment of Insulin Resistance; HR, hazard ratio; IRPI, intra-renal perfusion index; LV, left ventricular; MACE, major adverse cardiovascular events; MASLD, metabolic dysfunction-associated steatotic liver disease; MASPD, metabolic dysfunction-associated steatotic pancreas disease; MMP, matrix metalloproteinase; PI, perfusion index; PPV, positive predictive value; PRFT, perirenal fat thickness; RAAS, renin-angiotensin-aldosterone system; RRI, renal resistive index; SMI, superb microvascular imaging; Sn, sensitivity; Sp, specificity; SWE, shear wave elastography; T2DM, type 2 diabetes mellitus; TE, transient elastography; TIMP, tissue inhibitor of metalloproteinase; TyG, triglyceride-glucose; VAT, visceral adipose tissue.

## Bridging imaging and biochemistry: cross-parameter correlations in CRMS

EAT thickness at the posterior cardiac recess correlates with omental (r = 0.46) and perirenal fat (r = 0.58), tracking together across advancing metabolic syndrome severity alongside HOMA-IR (Di Gregorio et al., 2026).

The strongest replicated ultrasound-to-molecular correlation is between renal cortical echogenicity and serum creatinine/eGFR (r = 0.84–0.91) (Ahmed et al., 2019; Enamullah et al., 2023; Habtegiorgis et al., 2025). In the hepatic compartment, the attenuation coefficient in combination with ALT and INR can predict the presence of metabolic dysfunction-associated steatohepatitis, with an AUC of 0.85 (Liu et al., 2024). Combining liver stiffness measurement with FIB-4 improves fibrosis detection AUC from 0.83 to 0.92 compared with either parameter alone (Nishanov et al., 2025).

Carotid plaque grey-scale median correlates with circulating MMP-9, MMP-3, and TIMP-1, reflecting extracellular matrix remodeling. A low grey-scale median value combined with elevated MMP-9 was shown to predict major adverse cardiovascular events (Jiao et al., 2020; Kadoglou et al., 2023). Moreover, Gore et al. demonstrated that, when combined, the carotid imaging finding, NT-proBNP, hs-cTnI, and hs-CRP can stratify a three-year cardiovascular risk with an eight-fold gradient between highest and lowest scoring patients (Gore et al., 2020). The integration of cIMT into a composite vascular age model improved ten-year stroke and CVD risk prediction by 18% over the Framingham Risk Score (Maganti et al., 2025).

No published study has directly tried to associate specific ultrasound parameter values with the AHA/ACC CRMS stages (Khan et al., 2023). There have also been no studies that evaluated a unified pan-organ ultrasound protocol across carotid, cardiac, hepatic, and renal compartments simultaneously. Therefore, no validated protocol for such assessment exists.

## Emerging techniques: contrast-enhanced ultrasound and microvascular imaging in CRMS nephropathy phenotypes

Contrast-Enhanced Ultrasound (CEUS), using intravascular microbubbles, allows for real-time quantification of renal cortical microperfusion. The renal cortical perfusion index (PI) is significantly lower in CKD patients than in healthy controls (1618 vs. 3176 arbitrary units; p = 0.034) and correlates with eGFR (r = 0.54), while mean transit time is prolonged in CKD (3.6 s) compared to healthy individuals (1.8 s; p < 0.001), reflecting capillary rarefaction that precedes conventional laboratory deterioration (Garessus et al., 2021; Horne et al., 2025). In the field of CRMS nephropathy phenotypes, PI is lower in vascular than in diabetic nephropathy–which cannot be determined by conventional B-mode and Doppler imaging (Garessus et al., 2021).

Superb microvascular imaging (SMI) and related microvascular imaging (MVI) modalities detect low-velocity capillary flow without the need for contrast agents. The SMI vascular index correlates with eGFR (r = 0.56) and biopsy-confirmed fibrosis across CKD stages 2–5, outperforming conventional color Doppler (Armaly et al., 2022; Mao et al., 2022). In the cardiorenal context directly relevant to CRMS, the SMI-derived intra-renal perfusion index (IRPI) predicts congestion resolution and composite cardiovascular endpoints in acute decompensated heart failure independently of creatinine (AUC 0.73), capturing the hemodynamic-microvascular interface that underlies the cardiorenal phenotype of CRMS stage 3–4 (Kayama, 2024; 2025).

Together, CEUS and SMI have the potential to complement the multiparametric assessment described in Section *Renal parameters: resistive index, cortical echogenicity, and elastography* by evaluating the microvascular dimension and may 1 day enable non-invasive discrimination of CRMS nephropathy phenotypes (Querfeld et al., 2020).

## Toward an integrated multiparametric ultrasound screening protocol for CRMS

Before formulating such a protocol, three questions need to be answered to ensure it is deemed clinically useful and feasible: what to measure, how to guide the clinical decision-making with the measurements, and who can perform such measurements?

Based on the reviewed evidence, the CRMS assessment using multiparametric ultrasound should incorporate the following:

- B-mode hepatic assessment with steatosis and fibrosis grading, either with a Fibroscan or a 2D-SWE and attenuation assessment (Eddowes et al., 2019; Cao et al., 2022).
- Visceral fat measured as pre-peritoneal and omental fat thickness at the L4 level (Di Gregorio et al., 2026).
- EAT thickness measured on the right ventricular free wall, using transthoracic echocardiography (Iacobellis, 2023).
- cIMT and carotid plaque evaluation at the common carotid artery bifurcation with B-mode ultrasound (Johri et al., 2020; Kousios et al., 2020).
- For the kidneys, B-mode morphology and cortical echogenicity grading, perirenal fat thickness measurement, resistive index by pulsed-wave Doppler, and renal shear wave elastography where available to enable phenotype-oriented nephropathy assessment aligned with the CKM nephropathy patterns described in Section *Renal parameters: resistive index, cortical echogenicity, and elastography* (Sutikno and Baskoro, 2020; Romano et al., 2022; Bubnov, 2023; Stevens et al., 2024).

**FIGURE 1:**
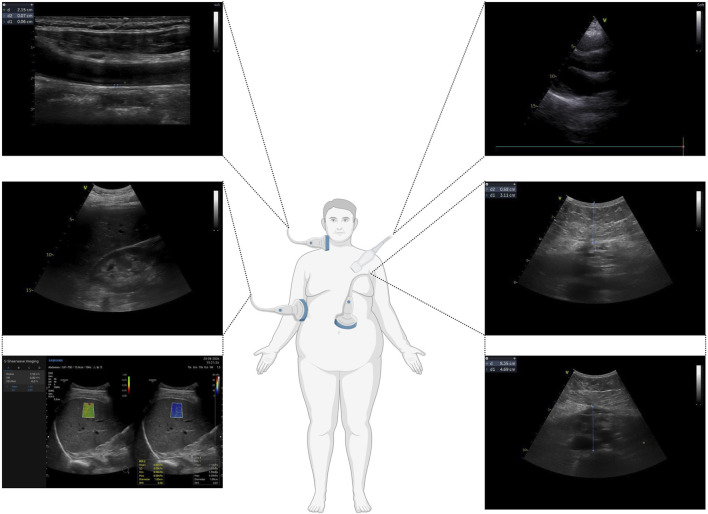
Proposed multiparamatric ultrasound protocol for CRMS assessment. Probe placement areas and representative images. From top on the left: carotid artery, hepatic parenchyma - kidney cortex interface, shear wave elastography of the liver. From top on the right: parasternal long axis of the heart, abdominal wall layers thickness measurement, omental fat thickness measurement.

**FIGURE 2:**
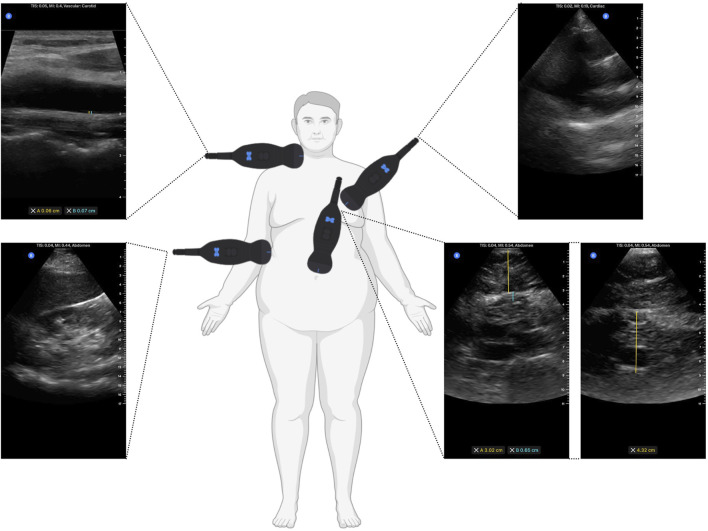
Proposed multiparamatric ultrasound protocol for CRMS assessment using a single, hand-held probe. Probe placement areas and representative images. From top on the left: carotid artery, hepatic parenchyma - kidney cortex interface. From top on the right: parasternal long axis of the heart, abdominal wall layers thickness measurement, omental fat thickness measurement.

The protocol should be constructed around the AHA/ACC CKM framework. It is worth noting that any abnormality found by the proposed protocol would automatically move a patient from Stage 0 into Stage 3 and thus warrant prompt intervention, i.e. with high-intensity lipid-lowering therapy and stricter LDL targets (Ndumele et al., 2023b). The probe placement areas of such a protocol are illustrated in Figure 1, with its POCUS feasibility shown in Figure 2.

Nowadays, the image quality of pocket-size ultrasound probes (Butterfly iQ3, VScan, Clarius, etc.) allows for the focused assessment of carotid arteries, cardiac chambers, abdominal fat, and renal cortex without the need for high-end equipment (Martinez et al., 2023; Kebrič et al., 2025). Andersen et al. showed that general practitioners can achieve competence in focused ultrasound examinations after two to thirty-one hours of structured hands-on training (Andersen et al., 2019). After a five-step course, family physicians were able to detect carotid atherosclerosis with 96.3% sensitivity and 90.0% specificity compared to vascular specialists (Kebrič et al., 2025). Furthermore, POCUS carotid assessment reclassified 39% of patients from high to remarkably high cardiovascular risk, directly altering therapeutic management (Kebrič et al., 2025). The accelerated diagnostic pathway may not only shorten the time from the evaluation into a therapeutic decision but also reduce the total cost of treatment, as has been shown for other in-hospital POCUS applications (Maganti et al., 2025).

## Discussion

The evidence reviewed confirms that multiparametric ultrasound assessment is not merely an anatomical evaluation but serves as a set of functional imaging biomarkers directly reflecting the molecular pathophysiology of CRMS. Nonetheless, some controversies remain to be solved before the wide adoption of such assessment. For carotid assessment, current ESC guidelines do not recommend routine cIMT measurement for cardiovascular risk stratification in the general population, citing only marginal net reclassification improvement over Framingham scoring—yet in CKD populations, cIMT independently predicts mortality beyond eGFR and albuminuria, suggesting that guideline recommendations developed for general populations may underestimate cIMT utility in CRMS-specific cohorts (Roumeliotis et al., 2019; Kousios et al., 2020). Transient elastography remains the most widely adopted and guideline-endorsed method, but its visco-elastic measurement properties cause overestimation in active hepatic inflammation, a condition prevalent in CRMS patients, whereas 2D-SWE isolates the elastic component and is less confounded (Mendoza et al., 2021). Whether TE cutoffs established in viral hepatitis populations are directly transferable to MASLD remains debated (Eddowes et al., 2019). Additionally, a reference standard modality for measuring epicardial adipose thickness needs to be established, as echocardiographic thickness and CT-derived volumetric or fat attenuation index assessments do not always correlate (Trimarchi et al., 2025). Across the parameters reviewed, the validated utility varies substantially and shall be considered when interpreting the proposed protocol. Liver stiffness measurement and CAP represent guideline-endorsed tools with established cutoffs and proven reproducibility across multicenter cohorts and hence could be considered ready for routine clinical application in CRMS assessment (Eddowes et al., 2019; Petroff et al., 2021). Renal SWE, perirenal fat thickness, and carotid plaque assessment represent promising biomarkers with growing evidence but lacking standardized acquisition protocols, vendor-independent cutoffs, or prospective outcome validation—therefore, it is our belief that they should currently be considered for specialized centers with research and tertiary-level infrastructure. Integrated multiparametric CRMS phenotyping models, CEUS-based renal perfusion assessment, and SMI microvascular examination remain exploratory tools requiring prospective multicenter validation before routine clinical adoption can be recommended.

Before translating the ultrasound use into clinical practice, a few gaps in the available literature are to be filled. No published study has established specific sonographic parameter values, i.e., cIMT thresholds, liver stiffness cutoffs, or RRI values to the AHA/ACC CRSM stages 0 through 4 in a prospective cohort (Khan et al., 2023). Secondly, no study was performed to assess the effect of multiparametric metabolic ultrasound screening in CRMS populations on clinical endpoints, such as mortality, end-stage kidney disease, and cardiovascular events. The composite model evidence reviewed in Section *Bridging imaging and biochemistry: cross-parameter correlations in CRMS* provides proof of concept but falls short of the prospective cohort data needed to justify guideline incorporation.

A shift from organ-oriented assessment should be made toward integrated system-based phenotyping. The cross-compartmental correlations reviewed in Section *Bridging imaging and biochemistry: cross-parameter correlations in CRMS*, namely epicardial fat tracking perirenal fat and HOMA-IR across metabolic syndrome severity (Cho et al., 2025), liver stiffness predicting incident CKD (Jung et al., 2024), and carotid plaque grey-scale median correlating with circulating MMPs (Kadoglou et al., 2023), demonstrate that no single parameter captures the full CRMS phenotype and that imaging-biochemistry composite models have the potential to outperform single-parameter approaches. A precision medicine framework would link each ultrasound parameter to its molecular axis: hepatic CAP and LSM to HOMA-IR and adipokine dysregulation; EAT thickness to IL-17A and hs-CRP; cIMT to osteopontin and eGFR trajectory; renal RI and renal SWE to tubular injury biomarkers (NGAL, KIM-1) and cystatin C; and perirenal adipose tissue to leptin and TNF-α (Demir et al., 2019; Roumeliotis et al., 2019; Qiu et al., 2024; Romano et al., 2025; Di Gregorio et al., 2026). This would enable non-invasive patient stratification by the dominant pathophysiological pathway rather than by organ alone. Translating this framework into clinical practice will require prospective cohort studies mapping specific ultrasound and biochemical parameter values to AHA/ACC CKM stages 0–4, outcome-based validation of the multiparametric protocol against hard endpoints, and standardization of acquisition protocols across equipment platforms.

AI-assisted image analysis represents a promising tool to simplify CRMS ultrasound assessment. Deep learning models applied to renal ultrasonography have already demonstrated accuracy for CKD staging and fibrosis quantification comparable to expert sonographers, raising the prospect of automated, simultaneous multiparametric CRMS phenotyping at the point of care that could return a structured organ-risk profile alongside the conventional biochemistry (Alnazer et al., 2021; Sabanayagam et al., 2025). However, such AI-augmented assessment will require multicenter validation across diverse populations and standardized image acquisition protocols universal across various vendors before such a solution could be recommended for routine clinical adoption.
