# Supplementary material for: Beyond biochemistry: multiparametric ultrasound parameters and their molecular correlates in cardio-renal-metabolic syndrome
Source: Acta Biochim Pol. 2026 Jul 14;73:16873. doi: 10.3389/abp.2026.16873 (PMC13407380; doi:10.3389/abp.2026.16873)
Supplement: Supplementary file 1 [file Supplementaryfile1.docx]

**Supplementary File S1: Search Methodology**

**1. Overview of Search Strategy**

A narrative search of PubMed and the Consensus AI-assisted academic search engine (consensus.app) was conducted covering publications from January 2015 to April 2026, with selected searches narrowed to 2018 for molecular biology topics where recent evidence predominates. The search was organized by manuscript section using PICO-structured queries, with each query targeting a specific organ compartment, molecular domain, or clinical question addressed in the review.

Two complementary query types were formulated for each major topic:

- Technique-oriented queries: focusing on the ultrasound method as the intervention
- Phenotype-oriented queries: focusing on the clinical population or molecular outcome as the target

This dual-query approach was designed to mitigate the risk of systematic directional bias in AI-assisted retrieval, ensuring that evidence was identified from both an imaging and a clinical/molecular perspective for each topic.

**2. Inclusion and Exclusion Criteria**

***Inclusion criteria***

- Peer-reviewed publications in English
- Original research articles, systematic reviews, meta-analyses, and clinical practice guidelines
- Studies in adult human populations or animal models with metabolic syndrome, obesity, type 2 diabetes mellitus, chronic kidney disease, cardiovascular disease, or cardio-renal-metabolic syndrome
- Studies reporting quantitative ultrasound-derived parameters, molecular biomarkers, or clinical outcomes relevant to the PICO questions
- Conference abstracts included only when reporting data not available in peer-reviewed full-text publications, particularly for novel techniques (e.g., renal SWE in post-COVID nephropathy, CEUS in CKM nephropathy phenotypes)

***Exclusion criteria***

- Case reports and case series
- Non-quantitative narrative reviews without systematic methodology
- Paediatric populations (age <18 years)
- Publications not available in English

**3. AI Tool Verification Procedures**

The following verification steps were applied to all references identified through AI-assisted search tools.

***3.1 Consensus (consensus.app)***

- All candidate references returned by Consensus were independently cross-checked against PubMed records to confirm author names, journal, year of publication, volume, issue, and DOI
- Full-text screening was performed by the primary author for all studies cited in the manuscript; only sources for which the primary document was reviewed and confirmed to support the attributed claim were retained
- Where Consensus results returned conference abstracts, the abstract text was retrieved directly from the publisher platform (Oxford Academic NDT, Frontiers) to verify data accuracy before citation
- Reviewer-suggested references (three URLs provided by Reviewer 1) were retrieved in full text and verified independently of the Consensus search results

***3.2 NotebookLM***

- NotebookLM was used as a secondary retrieval system to cross-query the collected PDF library, which was built using Zotero or by manual retrieval
- NotebookLM outputs were used to identify specific data points (correlation coefficients, cutoff values, AUROCs) for cross-checking against the source PDFs before inclusion
- NotebookLM citations were verified against the original PDF annotations in Zotero before use

***3.3 Claude Sonnet 4.6 (Anthropic)***

- Claude Sonnet 4.6 was used to support manuscript drafting and editing; it was not used as a primary literature retrieval tool
- All factual claims in AI-assisted text were verified against the cited source documents before approval
- All content generated with AI assistance was critically reviewed, revised for scientific accuracy, and approved by primary author prior to submission

***3.4 Limitations***

The narrative nature of this search precludes a formal assessment of completeness or impartiality equivalent to that of a systematic review with PRISMA reporting. The dual PICO query strategy and multi-tool cross-verification described above were employed to mitigate — but not eliminate — the risk of directional bias inherent to AI-assisted retrieval. Readers are encouraged to treat this review as a synthesized narrative overview rather than a comprehensive systematic assessment of the evidence base.

**4. Table S1: Consensus Search Query Strings**

Queries are listed in order of manuscript section. Mode indicates the Consensus search mode used (Basic, Pro, Deep Review, or Medical — the latter used during revision-phase searches). Revision-phase queries (§ Rev.) were conducted during peer review to address specific reviewer requests for expanded renal and emerging techniques content.

| **Section** | **Query string** | **Tool** | **Mode** | **Year filter** |
| --- | --- | --- | --- | --- |
| §2.1 | What are the key adipokine abnormalities in cardio-renal-metabolic syndrome and how do leptin, adiponectin and resistin relate to insulin resistance and inflammation? | Consensus | Pro | 2018–2025 |
| §2.2 | What is the role of chronic low-grade inflammation — IL-6, TNF-α, CRP, and pentraxin-3 — in the progression of cardiorenal metabolic syndrome? | Consensus | Pro | 2018–2025 |
| §2.3 | How do monocyte and lymphocyte subsets and macrophage polarization contribute to cardiovascular and renal injury in metabolic syndrome? | Consensus | Basic | 2018–2025 |
| §3.1.1 | Does visceral fat thickness measured by ultrasound correlate with insulin resistance and cardiometabolic risk markers? | Consensus | Pro | 2015–2025 |
| §3.1.2 | How does liver stiffness measured by elastography correlate with inflammatory markers, adipokines and insulin resistance in MASLD or NAFLD? | Consensus | Deep Review | 2015–2025 |
| §3.1.3 | How accurate is controlled attenuation parameter (CAP) for grading hepatic steatosis and how does it compare to liver biopsy in metabolic syndrome patients? | Consensus | Pro | 2015–2025 |
| §3.1.4 | Does epicardial adipose tissue thickness measured by echocardiography or ultrasound correlate with adipokines, insulin resistance, and cardiovascular risk in metabolic syndrome? | Consensus | Deep Review | 2015–2025 |
| §3.1.5 | What is the relationship between pancreatic steatosis assessed by ultrasound and insulin resistance or beta-cell dysfunction? | Consensus | Pro | None |
| §3.2.1 | Is carotid intima-media thickness a reliable imaging biomarker of cardiovascular risk in patients with chronic kidney disease and metabolic syndrome? | Consensus | Deep Review | 2015–2025 |
| §3.2.2 | What is the role of epicardial adipose tissue as a local mediator of coronary inflammation and what cytokines does it secrete? | Consensus | Pro | 2018–2025 |
| §3.3.1 | Does renal resistive index measured by Doppler ultrasound predict GFR decline or albuminuria progression in chronic kidney disease? | Consensus | Deep Review | 2015–2025 |
| §3.3.2 | Can renal shear wave elastography detect kidney fibrosis and predict CKD progression? | Consensus | Deep Review | None |
| §3.3.3 | How does renal cortical echogenicity on ultrasound relate to kidney function and CKD staging? | Consensus | Basic | 2015–2025 |
| §4.1 | Do combined multiparametric ultrasound assessments outperform individual biochemical markers for cardiovascular and metabolic risk stratification? | Consensus | Deep Review | 2018–2025 |
| §4.2 | How do visceral fat, liver stiffness, epicardial fat and carotid IMT correlate with each other and with albuminuria, cystatin C and HOMA-IR in metabolic syndrome? | Consensus | Pro | 2018–2025 |
| §5.1 | What is the feasibility and diagnostic yield of a comprehensive point-of-care ultrasound protocol for cardiometabolic risk screening in primary care? | Consensus | Pro | 2018–2025 |
| §5.2 | How does the AHA/ACC 2023 CKM syndrome staging framework guide clinical screening and risk stratification? | Consensus | Basic | 2022–2025 |
| §6.1 | What do CEUS-derived renal cortical perfusion parameters reveal about microvascular disease and GFR decline in chronic kidney disease? | Consensus | Pro | None |
| §6.2 | Can contrast-enhanced ultrasound detect hepatic microvascular changes in MASLD or NAFLD? | Consensus | Basic | None |
| §6.3 | Can superb microvascular imaging or microflow imaging detect early hepatic or renal microvascular rarefaction in metabolic disease? | Consensus | Basic | None |
| §6.4 | How does MRI-derived pancreatic fat fraction compare to ultrasound echogenicity for quantifying pancreatic steatosis and predicting metabolic dysfunction? | Consensus | Pro | None |
| §3.3 Rev. | Renal shear wave elastography CKD fibrosis metabolic syndrome diabetic nephropathy | Consensus | Pro | 2020–2026 |
| §3.3 Rev. | Perirenal fat ultrasound thickness chronic kidney disease eGFR metabolic risk CKM | Consensus | Pro | 2020–2026 |
| §3.3 Rev. | Multiparametric renal ultrasound integrated protocol CKD phenotyping fibrosis Doppler elastography | Consensus | Pro | 2021–2026 |
| §3.3 Rev. | Renal SWE elastography confounders limitations perfusion hydration body habitus depth obesity | Consensus | Pro | 2019–2026 |
| §3.3 Rev. | Shear wave elastography kidney post-COVID renal fibrosis stiffness SARS-CoV-2 | Consensus | Pro | 2021–2026 |
| §3.3 Rev. | Multiparametric renal ultrasound phenotypes CKM nephropathy metabolic syndrome diabetic hypertensive gout integrated assessment | Consensus | Pro | 2020–2026 |
| §5 Rev. | Contrast enhanced ultrasound CEUS kidney renal perfusion CKD diabetic nephropathy microvascular rarefaction metabolic syndrome | Consensus | Pro | 2020–2026 |
| §5 Rev. | Superb microvascular imaging SMI kidney renal CKD fibrosis vascular index | Consensus | Pro | 2019–2026 |
| §5 Rev. | Post-COVID renal injury ultrasound kidney CKD SARS-CoV-2 microvascular damage | Consensus | Pro | 2021–2026 |

**5. Table S2: NotebookLM Query Strings**

The following queries were used to interrogate the uploaded PDF library in NotebookLM. Each query was directed at the section-specific notebook containing the relevant papers. Outputs were used to identify quantitative data points for cross-checking against source PDFs before inclusion in the manuscript.

| **Section / Topic** | **Query string** |
| --- | --- |
| Adipokines (§2.1) | Which papers report specific serum levels or ratios of leptin, adiponectin, and resistin in patients with metabolic syndrome, obesity, or CKD? What were the key findings and were any correlated with cardiovascular or renal outcomes? |
| Adipokines (§2.1) | What is the evidence across these papers for the leptin/adiponectin ratio as a composite marker of cardiometabolic risk? What cutoffs or thresholds are mentioned? |
| Adipokines (§2.1) | Which papers discuss visfatin or resistin specifically in the context of insulin resistance or kidney disease? What were the main findings? |
| Inflammation (§2.2) | Which papers report data on IL-6, TNF-α, CRP, or pentraxin-3 in patients with metabolic syndrome, MASLD, or CKD? What are the reported levels and their clinical associations? |
| Inflammation (§2.2) | What is the evidence for pentraxin-3 as a marker of cardiovascular risk in cardiometabolic patients? How does it compare to CRP across these papers? |
| Inflammation (§2.2) | Which papers discuss NF-κB pathway activation or inflammasome involvement in metabolic syndrome or CKD progression? |
| Insulin resistance (§2.3) | Which papers use HOMA-IR as a marker and correlate it with organ damage, cardiovascular risk, or kidney function? What HOMA-IR thresholds are cited? |
| Insulin resistance (§2.3) | What insulin resistance indices are used across these papers beyond HOMA-IR — such as TyG index, QUICKI, or fasting insulin — and how are they validated in cardiometabolic populations? |
| Kidney markers (§2.4) | Which papers report data on cystatin C, NGAL, or KIM-1 in early CKD or diabetic nephropathy? How do these markers compare to eGFR for early detection? |
| Kidney markers (§2.4) | What is the evidence across these papers linking albuminuria to cardiovascular risk beyond kidney disease? Which populations were studied? |
| Kidney markers (§2.4) | Which papers discuss the relationship between eGFR decline and inflammatory markers or adipokines in metabolic syndrome patients? |
| Immune dysregulation (§2.5) | Which papers report data on monocyte subsets or macrophage polarization in metabolic syndrome, obesity, or CKD? What are the main findings regarding M1/M2 balance? |
| Immune dysregulation (§2.5) | What is the evidence for lymphocyte subset changes — particularly regulatory T cells or CD8+ effector populations — in cardiovascular or renal injury in metabolic patients? |
| Immune dysregulation (§2.5) | Which papers discuss inflammasome activation, particularly NLRP3, in the context of CKD or metabolic syndrome? |
| Ectopic fat (§3.1) | Which papers report ultrasound measurements of visceral omental fat and correlate them with HOMA-IR, fasting glucose, or HbA1c? What correlation coefficients are reported? |
| Ectopic fat (§3.1) | Which papers measure epicardial adipose tissue by echocardiography and correlate it with specific inflammatory cytokines — particularly IL-17A, IL-6, TNF-α, or hs-CRP? |
| Ectopic fat (§3.1) | Which papers discuss pancreatic steatosis measured by ultrasound and its relationship to beta-cell function, insulin secretion, or HOMA-IR? |
| Integrative (§4) | Synthesize the main molecular mechanisms linking adipose tissue dysfunction to both cardiovascular disease and kidney injury across these papers. Which molecular pathways appear in more than three papers? |
| Integrative (§4) | Which papers describe composite models combining ultrasound and biochemical markers? What were the AUROCs or diagnostic accuracies reported? |

**Abbreviations**

AUC, area under the curve; AUROC, area under the receiver operating characteristic curve; CAP, controlled attenuation parameter; CEUS, contrast-enhanced ultrasound; CKD, chronic kidney disease; CKM, cardiovascular-kidney-metabolic; CRP, C-reactive protein; eGFR, estimated glomerular filtration rate; EAT, epicardial adipose tissue; HbA1c, glycated haemoglobin; HOMA-IR, Homeostatic Model Assessment of Insulin Resistance; IL, interleukin; IMT, intima-media thickness; KIM-1, kidney injury molecule-1; MASLD, metabolic dysfunction-associated steatotic liver disease; NAFLD, non-alcoholic fatty liver disease; NGAL, neutrophil gelatinase-associated lipocalin; NLRP3, NLR family pyrin domain containing 3; PICO, Population Intervention Comparison Outcome; PRISMA, Preferred Reporting Items for Systematic Reviews and Meta-Analyses; SWE, shear wave elastography; TNF-α, tumour necrosis factor alpha; TyG, triglyceride-glucose.
